# A reproducible pipeline for generating analysis-ready paddy-field segmentation datasets from cadastral vectors and Pléiades very-high-resolution imagery^☆^

**DOI:** 10.1016/j.mex.2026.104070

**Published:** 2026-07-25

**Authors:** Herman Setiadi, Muhammad Zarlis, Edy Irwansyah, Yulyani Arifin

**Affiliations:** a Computer Science Department, BINUS Graduate Program - Doctor of Computer Science, Bina Nusantara University, Anggrek Campus, Jl. Kebon Jeruk Raya No 27, Kebon Jeruk, Jakarta, 11530, Indonesia; b Information Systems Management Department, BINUS Graduate Program - Master of Information Systems Management, Bina Nusantara University, Anggrek Campus, Jl. Kebon Jeruk Raya No 27, Kebon Jeruk, Jakarta, 11530, Indonesia

**Keywords:** Remote sensing, Rasterization, Sliding windows, Radiometric normalization, Landscape metrics, Semantic labeling, Reproducibility

## Abstract

This method converts official Lahan Baku Sawah cadastral vectors and Pléiades very-high-resolution imagery into analysis-ready paddy-field segmentation tiles. It aligns coordinate reference systems, rasterizes polygons onto the reference image grid, tiles paired image and mask arrays using 1024 × 1024 windows with a 512-pixel stride, applies per-band 2nd-98th percentile normalization while excluding no-data pixels, removes tiles below 1% paddy coverage, and computes connected-component landscape descriptors. Applied to five Indonesian regions, the workflow produced 3108 image-mask pairs spanning a wide range of Largest Patch Index values (9.40–34.43%). An exploratory validation used a separate 48-tile metadata subset: a custom U-Net was trained on two tiles per region and evaluated on the remaining 38 tiles, while SAM 1 was evaluated with a ground-truth-derived bounding-box prompt. The results confirm that the exported PNG pairs can be consumed by standard segmentation pipelines, but the small, overlapping validation subset is not intended as a benchmark. The source TIFFs are publicly available on Figshare, and the processed dataset is available on Mendeley Data, Version 3.

• Produces co-registered 1024 × 1024 RGB image and binary-mask tiles from cadastral vectors and very-high-resolution imagery.

Handles coordinate-system mismatch, no-data borders, overlapping windows, coverage filtering, and normalization.

## Graphical abstract

Full-scene Pléiades RGB imagery and expert-produced LBS labels are aligned and rasterized, tiled with 50% overlap, normalized and filtered, then exported as 3108 analysis-ready image-mask pairs with landscape descriptors.

**Figure:**
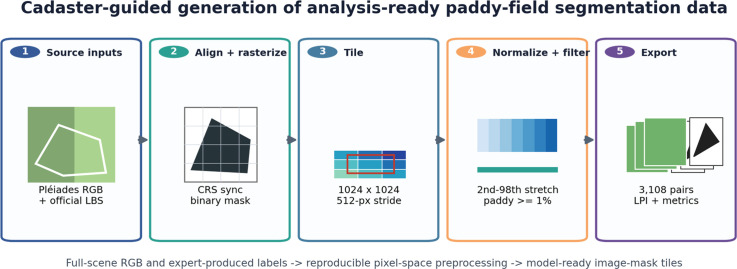

**Specifications table**

**Table:** 

**Subject area**	Computer Science
**More specific subject area**	Remote sensing; semantic segmentation of very-high-resolution satellite imagery
**Name of your method**	Cadaster-guided VHR segmentation-dataset generation
**Name and reference of original method**	The workflow is not a customization of one named method. It combines geospatial vector reprojection and rasterization with rasterio.features.rasterize [1], overlapping sliding-window tiling, valid-pixel percentile normalization, and connected-component landscape descriptors inspired by FRAGSTATS [2].
**Resource availability**	The public Figshare record *Raw Full-Scene Pléiades Imagery and Ground-Truth Masks for Paddy Field Segmentation across Five Agro-Ecological Zones in Indonesia* provides five unclipped, non-georeferenced _raw.tif rasters and five pixel-aligned _mask.tif master masks: https://doi.org/10.6084/m9.figshare.32938838 (CC BY 4.0) [3]. The processed dataset contains 3108 image-mask pairs, metadata, landscape metrics, and processing code: Mendeley Data, Version 3, https://doi.org/10.17632/g7vkrjr8dn.3 (CC BY 4.0; no registration required) [4]. The implementation uses Python 3.14 with rasterio [1], GeoPandas [5], Shapely, NumPy, OpenCV, and tqdm.

## Background

Semantic segmentation of smallholder paddy fields in tropical Asia is difficult because fields are often small, irregular, terraced, seasonally flooded, and embedded in heterogeneous land cover. These properties produce high within-class spectral variability and complex boundaries. Fragmentation of irrigated and rainfed paddy fields has been documented in West Java [6]. GEO-Bench provides broad Earth-monitoring tasks but is not dedicated to Indonesian smallholder paddy parcels [7]. Preparing model-ready data for such landscapes requires analysts to reconcile coordinate reference systems, rasterize cadastral polygons at the source-image resolution, handle very-high-resolution radiometry and no-data borders, and tile image-mask pairs consistently.

This article provides a deterministic workflow for those tasks. It accepts an authoritative paddy cadaster and pan-sharpened RGB ortho imagery, then produces uniform 1024 × 1024 image-mask pairs for conventional convolutional and prompt-based segmentation experiments. Remote-sensing segmentation under limited supervision provides relevant methodological context [8], while SAM 2 and geospatial foundation models motivate future adaptation studies [9,10]. The labels originate from the Indonesian Lahan Baku Sawah (LBS) cadastral vector information rather than model-generated annotation. The workflow also computes connected-component landscape descriptors inspired by FRAGSTATS [2] so that users can characterize morphological variation among regions and design spatially separated validation protocols. The method was developed with LBS polygons and Pléiades 1A/1B imagery [11] and demonstrated across five contrasting Indonesian regions. The derived tiles, metadata, metrics, and code are available in the Mendeley Data record [4].

## Method details

The workflow has two reproducibility levels. With originally georeferenced imagery and the LBS vector data, users can run the complete coordinate synchronization and rasterization stage. With the public Figshare TIFF pairs, users can reproduce the pixel-space tiling, normalization, filtering, export, and landscape-descriptor stages. The Figshare copies intentionally omit absolute geolocation; therefore, they cannot reproduce the initial vector-to-raster alignment or be overlaid on external geospatial layers.

### Software and source inputs

The implementation uses Python 3.14 with rasterio [1], GeoPandas [5], Shapely, NumPy, OpenCV, and tqdm.

The public Figshare record contains one unclipped, non-georeferenced raw raster and one pixel-aligned binary master mask for each region:

• BANGLI_SUSUT_raw.tif and BANGLI_SUSUT_mask.tif.
• KULONPROGO_GALUR_raw.tif and KULONPROGO_GALUR_mask.tif.
• LEUWIDAMAR_raw.tif and LEUWIDAMAR_mask.tif.
• SITUBONDO_SITUBONDO_raw.tif and SITUBONDO_SITUBONDO_mask.tif.
• Tangerang_Sepatan_Timur_raw.tif and Tangerang_Sepatan_Timur_mask.tif.

The raw TIFFs preserve the unclipped pixel arrays used by the downstream pipeline but omit the absolute coordinate reference system and map origin because precise field locations are sensitive; only approximate site centroids are reported. The mask TIFFs have identical width and height to their paired raw raster and use 0 for background and 255 for paddy.

The masks are derived from the official LBS reference data. Paddy-field boundaries were manually delineated and verified by experts from the Indonesian Ministry of Agrarian Affairs and Spatial Planning (ATR/BPN) and the Ministry of Agriculture as part of national paddy-field mapping activities. They are therefore authoritative, expert-produced ground truth rather than model-generated annotations.

### Coordinate-reference synchronization and rasterization

For full reproduction with licensed, georeferenced inputs, the reference image is opened with rasterio [1], and its affine transform, coordinate reference system (CRS), width, height, and bounding box are recorded. The corresponding LBS GeoPackage is opened with GeoPandas [5]. If the vector and raster CRS values differ, the vector geometries are reprojected to the raster CRS. A bounding-box intersection check stops processing when the two layers do not overlap.

The LBS geometries are rasterized onto the reference grid with rasterio.features.rasterize, assigning 255 to paddy pixels and 0 to background pixels. The output is an 8-bit, pixel-aligned master mask. The released implementation must explicitly preserve the rasterization inclusion rule used to generate the deposited masks rather than rely on an unstated library default.

### Sliding-window tiling

The raw raster and its master mask are traversed using a 1024 × 1024-pixel window and a 512-pixel stride, giving 50% overlap in both directions. Image and mask windows use identical pixel coordinates. Overlap preserves fields that cross tile boundaries but also creates spatial dependence between neighboring tiles; region-disjoint or source-disjoint splits are therefore preferred for independent model evaluation.

### No-data-aware radiometric normalization

The source rasters store pan-sharpened RGB digital numbers in a 32-bit container and use 65,536 as the no-data sentinel. Valid values occupy approximately 0–4700. For each tile and band, pixels satisfying 0 < value < 50,000 are used to estimate the 2nd and 98th percentiles. Values are clipped to that interval and linearly scaled to 0–255. Pixels outside the validity mask are set to 0 after scaling. This prevents no-data borders from dominating the percentiles and collapsing the visible contrast of partially off-scene tiles. The released code should retain an explicit fallback for tiles or bands with no valid pixels or identical percentile values.

### Coverage filtering and export

Paddy coverage is calculated as the proportion of mask pixels equal to 255 in the full 1024 × 1024 window. Tiles with <1% paddy coverage are discarded. Retained images are exported as 8-bit RGB PNG files, and masks are exported as 8-bit single-channel PNG files with values 0 and 255.

### Landscape-descriptor computation

Connected components are identified in each retained binary mask using 8-connectivity. Patch area is measured in pixels. The following per-tile descriptors are calculated and then averaged within each region:

• Number of patches (NP): the number of paddy components.
• Mean patch size (MPS): the arithmetic mean component area in pixels.
• Largest Patch Index (LPI): largest paddy component area divided by total tile area, multiplied by 100.
• Patch density (PD): NP per megapixel of total tile area.
• Edge density (ED): summed perimeter of external paddy contours in pixels, divided by total tile area.
• Fragmentation index: regional mean ED divided by regional mean MPS.

LPI uses the standard landscape-area denominator defined for the FRAGSTATS Largest Patch Index [2].

### Method output and dataset organization

Each region produces paired folders:

• images/: 1024 × 1024, 8-bit RGB PNG tiles.
• masks/: 1024 × 1024, 8-bit single-channel PNG masks (0 = background; 255 = paddy).

**Fig. 1:**
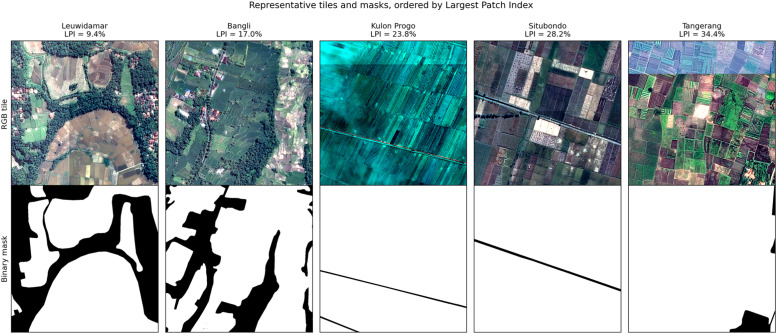
Representative RGB tiles (top row) and paired binary masks (bottom row), ordered by ascending regional mean LPI: Leuwidamar (9.40%), Bangli (17.00%), Kulon Progo (23.79%), Situbondo (28.24%), and Tangerang (34.43%). White pixels indicate paddy.

**Fig. 2:**
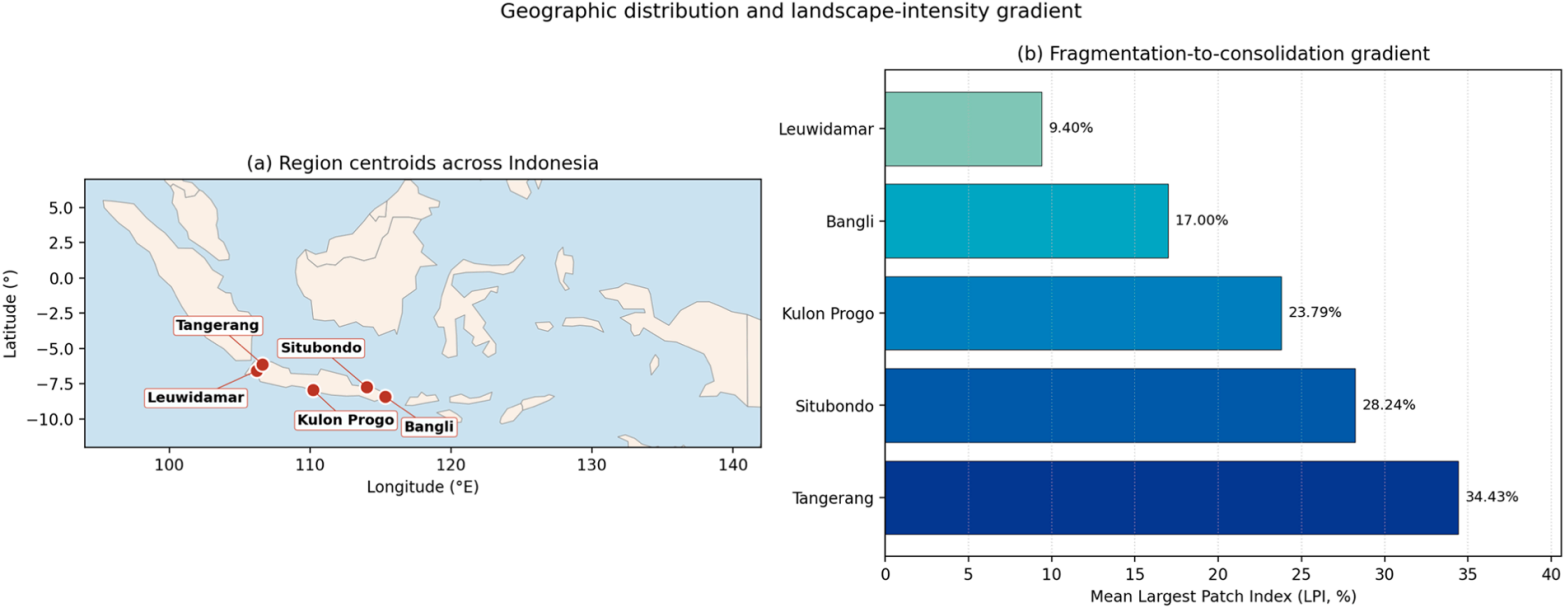
Approximate geographic distribution of the five study regions (left) and regional mean Largest Patch Index (LPI; right). Map lines delineate study areas and do not necessarily depict accepted national boundaries.

**Fig. 3:**
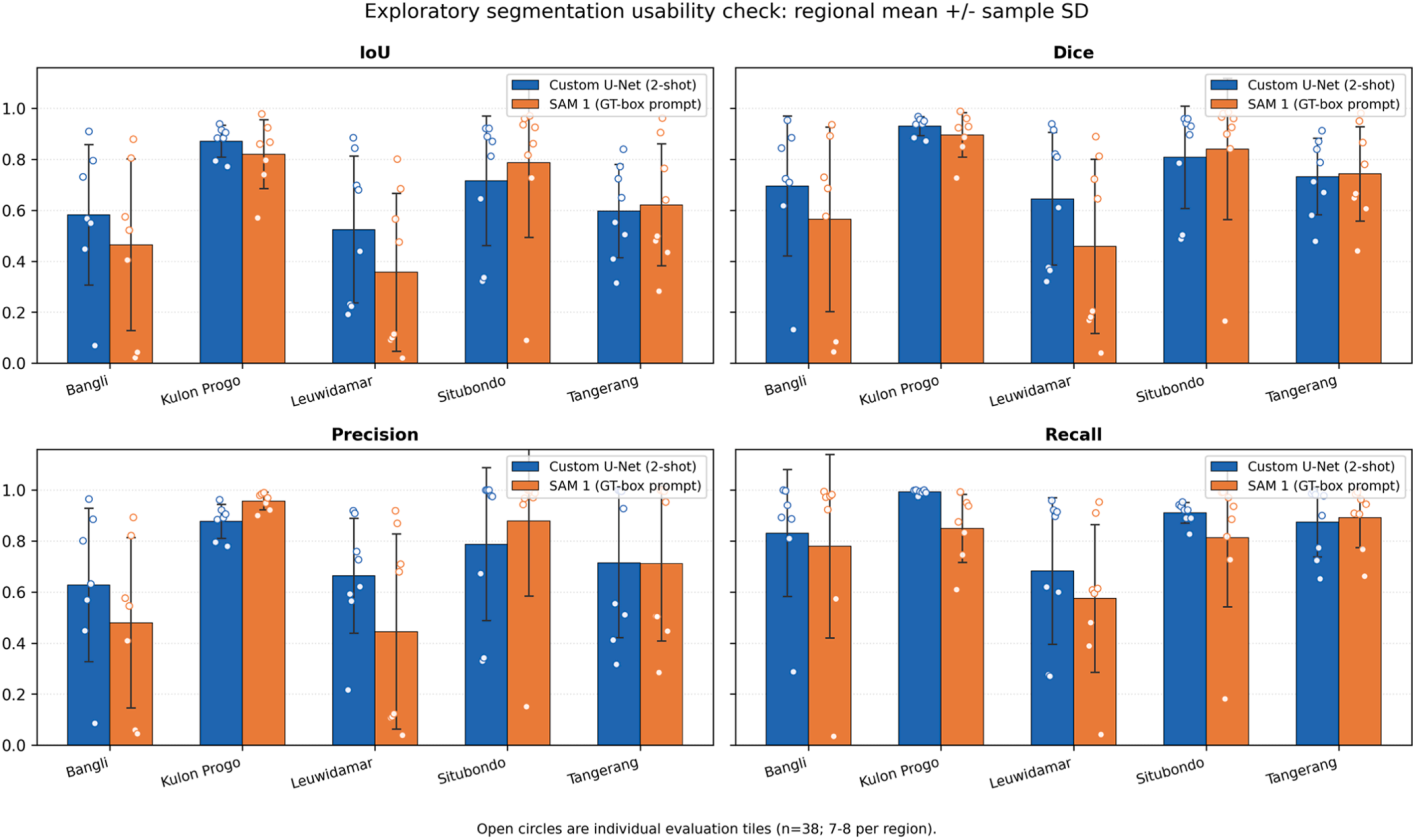
Per-region IoU, Dice, precision, and recall for the exploratory 38-tile evaluation. Bars show regional means, error bars show ±1 sample SD, and open circles show individual tiles (n = 7 for Bangli and Kulon Progo; n = 8 for Leuwidamar, Situbondo, and Tangerang). SAM 1 uses ground-truth-derived bounding-box prompts. Because the tiles overlap by 50%, the observations are spatially dependent; the standard deviations describe tile-level spread, not confidence intervals or training-seed uncertainty.

**Fig. 4:**
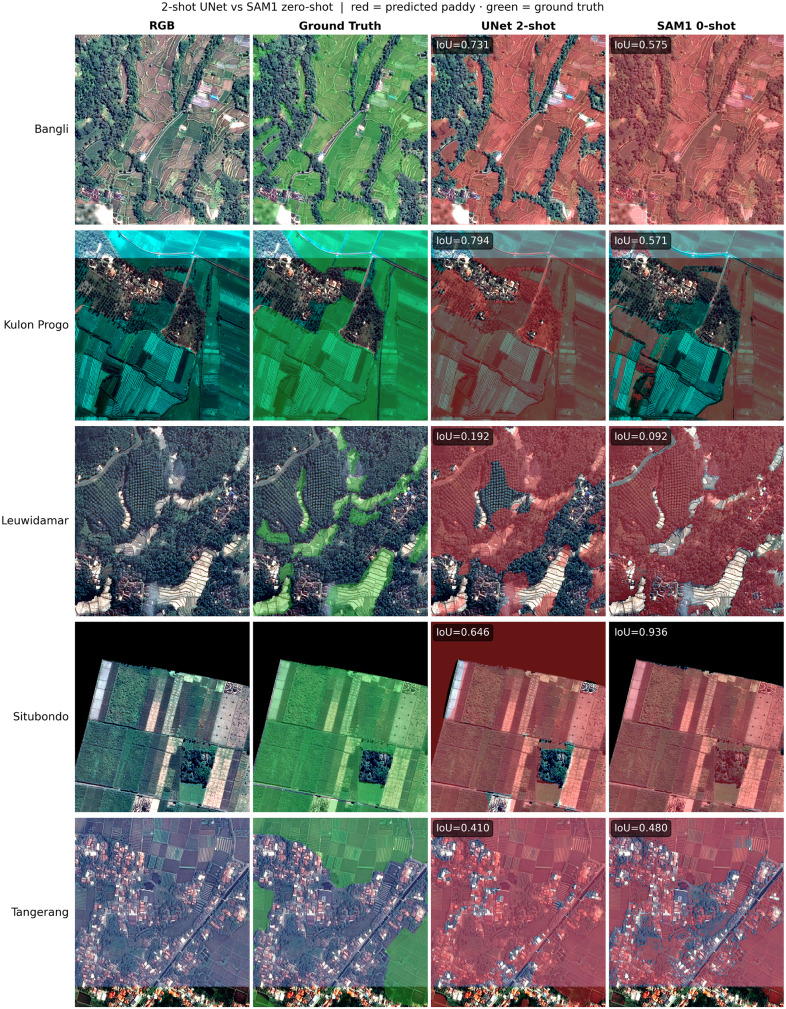
Illustrative evaluation tiles for each region. Columns show the RGB tile, ground-truth paddy mask (green), custom U-Net prediction (red), and SAM 1 prediction from a ground-truth-derived bounding box (red). IoU is printed in each prediction panel. These examples are descriptive and were not randomly sampled for statistical inference.

Tile names follow tile_{x}_{y}.png, where x and y are the top-left source-pixel coordinates. Matching names in images/ and masks/ identify co-registered pairs. The Mendeley Data Version 3 record [4] contains 3108 pairs across the five folders shown in Table 1, together with metadata, landscape metrics, and processing code. Use that processed record for model training. Use the public Figshare full-scene record to re-tile the scenes, inspect full-scene context, or apply an alternative preprocessing pipeline. Figs. 1,2,3,4

**Table 1:** Regional tile folders and dominant morphology.

**Folder**	**Region**	**Tiles**	**Dominant morphology**
sam_dataset_BANGLI_SUSUT	Bangli, Bali	495	Terraced, fragmented
sam_dataset_KULONPROGO_GALUR	Kulon Progo, Yogyakarta	447	Continuous traditional rice belt
sam_dataset_LEUWIDAMAR	Lebak, Banten	1536	Sparse mixed agroforestry
sam_dataset_SITUBONDO_SITUBONDO	Situbondo, East Java	325	Consolidated large blocks
sam_dataset_Tangerang_Sepatan_Timur	Tangerang, Banten	305	Peri-urban mosaic

### Method validation

Validation addresses two questions: whether the selected regions differ in mask morphology and whether the exported PNG pairs can be consumed by conventional and prompt-based segmentation code without additional format conversion. The model experiment is an exploratory usability check, not a comparative benchmark.

### Regional landscape descriptors

Table 2 summarizes the per-tile connected-component descriptors. Mean LPI ranges from 9.40% in Leuwidamar to 34.43% in Tangerang, showing substantial morphological variation among the five regional subsets. Because the values are regional summaries of overlapping tiles, they should not be interpreted as independent landscape samples.

**Table 2:** Region-level landscape descriptors (arithmetic mean over retained tiles). NP = number of patches; MPS = mean patch size in pixels; LPI = largest paddy patch divided by total tile area; PD = patch density per megapixel; ED = summed external-contour perimeter divided by total tile area.

**Region**	**Retained tiles**	**Avg NP**	**Avg MPS**	**Avg LPI (%)**	**Avg PD**	**Avg ED**	**Fragmentation index**
BANGLI_SUSUT	495	5.58	65,579	17.00	5.32	6.04e-3	9.22e-8
KULONPROGO_GALUR	447	3.92	119,318	23.79	3.74	5.09e-3	4.27e-8
LEUWIDAMAR	1536	3.66	45,571	9.40	3.49	3.66e-3	8.03e-8
SITUBONDO_SITUBONDO	325	2.70	187,238	28.24	2.58	3.75e-3	2.00e-8
Tangerang_Sepatan_Timur	305	3.07	203,100	34.43	2.93	4.96e-3	2.44e-8

### Exploratory segmentation usability check

The available validation files contain a 48-tile metadata subset rather than all 3108 dataset tiles. With random seed 42, two tiles per region (10 total) were selected for U-Net training and recorded in 2shot_unet_train_tiles.csv; the remaining 38 tiles (7–8 per region) were evaluated.

The convolutional model is a custom four-level U-Net [12], not the ResUNet-a architecture. Images and masks were resized from 1024 × 1024 to 512 × 512 pixels for training. The model was trained for 10 epochs with batch size 2, Adam at a learning rate of 1e-4, and an equally weighted binary-cross-entropy and Dice loss. Predictions were thresholded at 0.5 and resized to the original tile dimensions with nearest-neighbor interpolation.

SAM 1 used the facebook/sam-vit-base checkpoint [13] without weight adaptation. Each prompt was a single bounding box derived from the corresponding ground-truth mask. This is an oracle-prompt usability test, not prompt-free zero-shot segmentation and not a fair performance comparison with the U-Net. IoU, Dice, precision, and recall were computed per tile. Table 3 reports mean and sample standard deviation for IoU and Dice; the complete per-tile metrics are provided with the validation CSV files.

**Table 3:** Exploratory segmentation results on 38 evaluation tiles (mean ± sample SD).

**Region**	**n**	**Custom U-Net IoU**	**Custom U-Net Dice**	**SAM 1 IoU**	**SAM 1 Dice**
Bangli	7	0.582 ± 0.276	0.696 ± 0.274	0.465 ± 0.336	0.564 ± 0.362
Kulon Progo	7	0.871 ± 0.063	0.930 ± 0.037	0.820 ± 0.135	0.896 ± 0.087
Leuwidamar	8	0.524 ± 0.288	0.645 ± 0.260	0.357 ± 0.310	0.458 ± 0.341
Situbondo	8	0.715 ± 0.254	0.808 ± 0.201	0.786 ± 0.293	0.841 ± 0.277
Tangerang	8	0.596 ± 0.183	0.732 ± 0.150	0.622 ± 0.239	0.743 ± 0.185

## Limitations

• The public Figshare record provides pixel-aligned full-scene raster–mask pairs. Their CRS, affine transform, and absolute coordinates were removed to protect sensitive locations; therefore, they support downstream pixel-space processing but not geographic overlay or reproduction of the original LBS vector alignment.
• LBS polygons represent cadastral paddy extent; transiently fallow or converted parcels may remain labeled as paddy. No independent multi-annotator label-quality audit is currently reported.
• Acquisition dates are not aligned across regions, so phenology and regional identity may be confounded. The manuscript does not yet report per-scene acquisition metadata or the temporal gap between imagery and LBS vintage.
• The 50% tile overlap creates spatial dependence. Random tile-level train/test splits can leak near-duplicate spatial context; source- or region-disjoint splits should be used for independent evaluation.
• The segmentation validation covers only 38 evaluation tiles from a 48-tile subset. It does not support population-level performance claims for all 3108 pairs. The U-Net result uses one random seed, and SAM 1 receives a privileged ground-truth-derived bounding box.
• The delivered imagery contains three visible bands only. It is therefore better suited to spatial-pattern and boundary-segmentation tasks than to multispectral or multi-temporal phenology analysis.

## Ethics statements

The work does not involve human participants, animal experiments, human biological material, or data collected from social media platforms.

## CRediT author statement

Herman Setiadi: Conceptualization, Methodology, Software, Data curation, Writing - original draft. Muhammad Zarlis: Supervision, Writing - review and editing. Edy Irwansyah: Supervision, Validation, Writing - review and editing. Yulyani Arifin: Supervision, Validation, Writing - review and editing.

## Declaration of generative AI and AI-assisted technologies in the manuscript preparation process

During the preparation of this work, the authors used OpenAI Codex to support language refinement, consistency checking, and formatting against the MethodsX template. After using this service, the authors reviewed and edited the content as needed and take full responsibility for the content of the published article.

## Declaration of competing interest

The authors declare that they have no known competing financial interests or personal relationships that could have appeared to influence the work reported in this paper.

## Data Availability

data has been publicly available

## Appendix Supplementary materials

Image, application 1

Image, application 2

Image, application 3

Image, application 4

Image, application 5

Image, application 6
